# Supplementary material for: Association between oral microbiome and seven types of cancers in East Asian population: a two-sample Mendelian randomization analysis
Source: Front Mol Biosci. 2023 Nov 21;10:1327893. doi: 10.3389/fmolb.2023.1327893 (PMC10702768; doi:10.3389/fmolb.2023.1327893)
Supplement: Supplementary file 1 [file DataSheet1.ZIP › Supplementary Materials/MR plots of saliva/saliva═╝/Prostate cancer/pheno.3098_to_prostate cancer_forest.pdf]

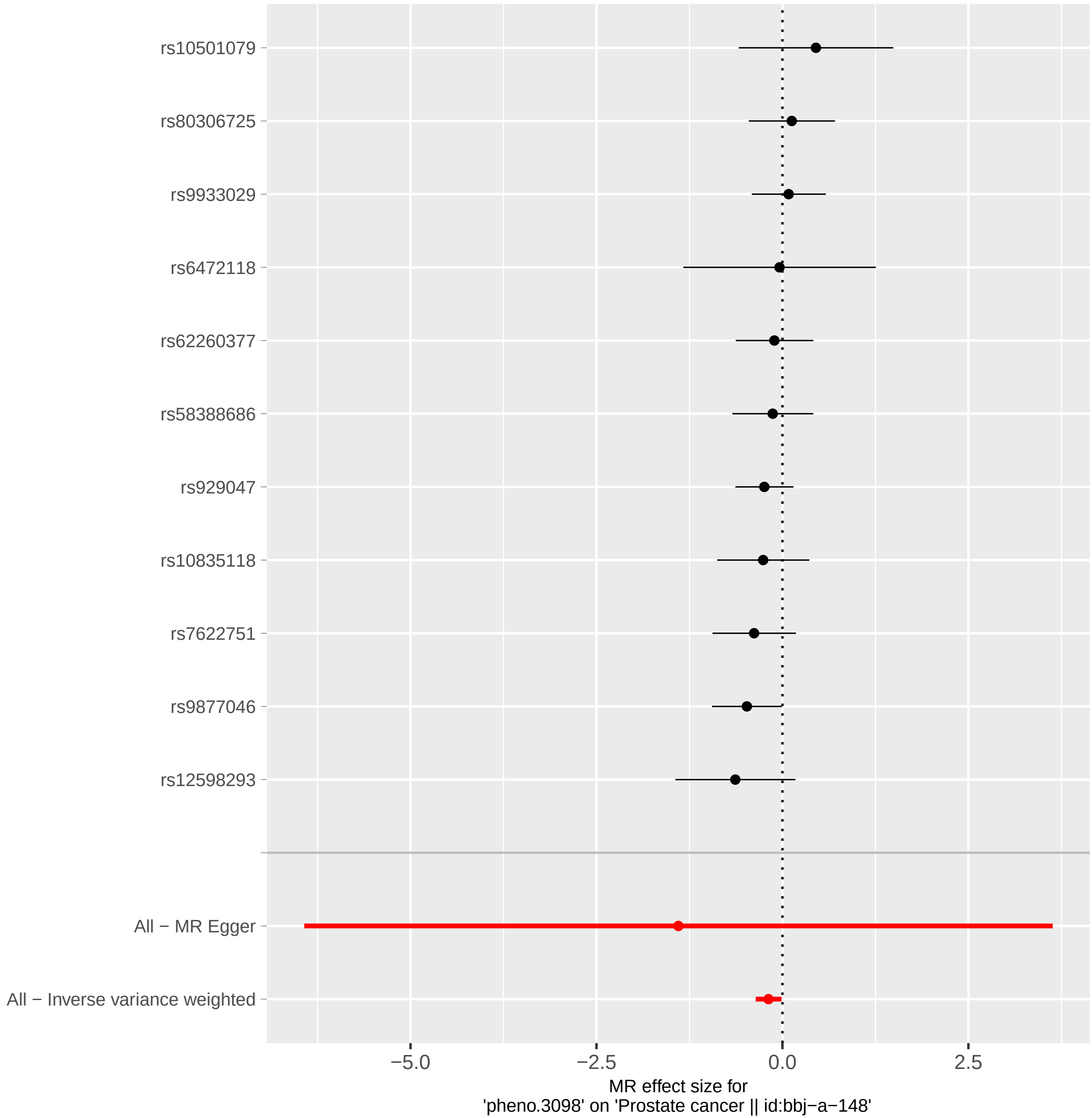

rs10501079
rs80306725
rs9933029
rs6472118
rs62260377
rs58388686
rs929047
rs10835118
rs7622751
rs9877046
rs12598293
All − MR Egger
All − Inverse variance weighted
−5.0
−2.5
0.0
2.5
MR effect size for
'pheno.3098' on 'Prostate cancer || id:bbj−a−148'
